# Nucleic Acid Sensing Pathways in DNA Repair Targeted Cancer Therapy

**DOI:** 10.3389/fcell.2022.903781

**Published:** 2022-04-26

**Authors:** Bingteng Xie, Aiqin Luo

**Affiliations:** ^1^ School of Life Science, Beijing Institute of Technology, Beijing, China; ^2^ Key Laboratory of Molecular Medicine and Biological Diagnosis and Treatment, Beijing Institute of Technology, Ministry of Industry and Information Technology, Beijing, China

**Keywords:** DNA damage and repair, DDR inhibitors, nucleic acid-sensing pathways, innate immunity, immunotherapy

## Abstract

The repair of DNA damage is a complex process, which helps to maintain genome fidelity, and the ability of cancer cells to repair therapeutically DNA damage induced by clinical treatments will affect the therapeutic efficacy. In the past decade, great success has been achieved by targeting the DNA repair network in tumors. Recent studies suggest that DNA damage impacts cellular innate and adaptive immune responses through nucleic acid-sensing pathways, which play essential roles in the efficacy of DNA repair targeted therapy. In this review, we summarize the current understanding of the molecular mechanism of innate immune response triggered by DNA damage through nucleic acid-sensing pathways, including DNA sensing *via* the cyclic GMP-AMP synthase (cGAS), Toll-like receptor 9 (TLR9), absent in melanoma 2 (AIM2), DNA-dependent protein kinase (DNA-PK), and Mre11-Rad50-Nbs1 complex (MRN) complex, and RNA sensing *via* the TLR3/7/8 and retinoic acid-inducible gene I (RIG-I)-like receptors (RLRs). Furthermore, we will focus on the recent developments in the impacts of nucleic acid-sensing pathways on the DNA damage response (DDR). Elucidating the DDR-immune response interplay will be critical to harness immunomodulatory effects to improve the efficacy of antitumor immunity therapeutic strategies and build future therapeutic approaches.

## 1 Overview of DNA Damage and Repair Network

Up until now, chemotherapy and radiotherapy have remained the important treatment options for a variety of cancers (Hellmann et al., 2016; Chu et al., 2018; Qin et al., 2018; De Ruysscher et al., 2019; Grassberger et al., 2019; Galluzzi et al., 2020; Huang and Zhou, 2020; McLaughlin et al., 2020). The key mechanism of tumor cell death induced by standard chemotherapy and radiotherapy is DNA damage, leading to cell-cycle arrest and death directly or after S-phase DNA replication in the cell cycle. On the other hand, to deal with possible DNA lesions, tumors cells have evolved intricate repair mechanisms, and the ability to repair therapy-induced DNA damage would influence the therapeutic efficacy (Bouwman and Jonkers, 2012; Gavande et al., 2016). Here, we first overview the various types of DNA damage caused by radiotherapy and chemotherapy and the corresponding DNA damage repair pathways (see previous reviews for details) (Ciccia and Elledge, 2010; Giglia-Mari et al., 2011; Pinder et al., 2013; Chatterjee and Walker, 2017; Cussiol et al., 2019; Souliotis et al., 2020).

Some commonly used potent chemotherapy compounds (cyclophosphamide, dacarbazine, cisplatin, etc.) act by adding the alkyl groups to specific bases of DNA, yielding alkylated products such as O2-alkylthymine, O4-alkylthymine, O6-methylguanine, and O6-ethylguanine (Serrone et al., 2000; Emadi et al., 2009; Dasari and Tchounwou, 2014). Monofunctional alkylating agents have one active moiety and can only modify a single base, while bifunctional alkylating agents have two reaction sites, which can crosslink DNA to protein or another DNA base resulting in intra-strand crosslinks or inter-strand crosslinks. Radiotherapy by ionizing radiation (IR) can attack DNA directly by breaking the phosphodiester bond and the deoxyribose, and other indirect means have also been confirmed, for example, highly reactive oxygen species (ROS) produced from water radiolysis could result in the oxidization of the DNA desoxyribose moiety and the four nitrogenous bases (Henner et al., 1982; Desouky et al., 2015). Thereby, chemotherapy and radiotherapy can result in various kinds of DNA damage including base damage, single-strand breaks (SSBs), and double-strand breaks (DSBs). Among them, DSBs are thought to be the most harmful to cell survival and are the main mechanism to promote the therapeutic effect.

To remedy various DNA damage types, there has developed a complex DNA damage response (DDR) network (Giglia-Mari et al., 2011). The pathways involved in DNA damage repair mainly include direct reversal, base excision repair (BER), nucleotide excision repair (NER), non-homologous end joining (NHEJ), and homologous recombination (HR) pathway. Direct reversal, the simplest DNA repair pathway, depends primarily on a single protein and does not involve nucleotide removal, resynthesis, or ligation. For example, the O6-alkyl group of guanines can be removed by O6-methylguanine DNA methyltransferase (MGMT). BER and NER pathways take part in the DNA SSBs repairment. BER often participates in the repair of the small but highly mutagenic DNA lesions, which usually significantly undermine genomic fidelity and stability (Dianov and Hübscher, 2013). The BER pathway is initiated with the excision of the damaged base by any of 11 DNA glycosylases (Krokan et al., 1997), after which the exposed gap will be filled by a different set of proteins, among which the repair of single-base gaps require the short-patch pathway while polybasic gaps are in need of the long-patch pathway (Woodrick et al., 2017; Biau et al., 2019; Caldecott 2020). The NER pathway involves multiple steps requiring more than 30 proteins and is the main pathway used by mammals to remove bulky DNA lesions, including numerous chemical adducts, intra-strand crosslinking of DNA, and some forms of oxidative damage. In the NER pathway, the damaged bases are first recognized, then the DNA double-strand is unwound, and then the excision repair complex will remove the damaged bases followed by filling and ligating of the gap (Gillet and Schärer, 2006; Shuck et al., 2008).

DSB is a highly toxic gene damage that seriously threatens cellular homeostasis by affecting the transcription of genes, DNA replication, and chromosome segregation. Failure in repairing DSBs can lead to devastating chromosomal instabilities, and result in the dysregulation of gene expression and an increased hazard of carcinogenesis (Kryston et al., 2011). In human cells, two pathways, HDR and NHEJ, take part in DNA DSBs repair (Chang et al., 2017). HDR is generally considered to be participated in DSBs repair only during the S and G2 phases of the cell cycle given that it needs a homologous template to replace the damaged DNA segment in the genome, but it has been shown that centromeric DSBs in the G1 phase can activate the HDR pathway to maintain centromeric integrity recently (Yilmaz et al., 2021). The canonical HDR pathway is relatively slow but error-free, which needs numerous factors involved in homology search, Holliday junction formation, DNA synthesis, and the final DNA ligation (San Filippo et al., 2008; Krejci et al., 2012). Unlike the HR pathway, the NHEJ pathway does not need a DNA template and is active throughout the whole cell cycle, therefore it responds relatively quickly but is error-prone (Chang et al., 2017). In NHEJ pathway, four specific steps are involved including DNA termini recognition, bridging of the DNA ends, DNA end processing, and DNA ligation.

## 2 DNA Repair Targeted Therapy

Given that tumor cells could repair DNA damage induced by chemotherapy and radiotherapy in order to survive, the use of inhibitors of specific DNA repair pathways combined with DNA-damaging treatment can be efficacious. Some DNA repair inhibitors have been exploited as clinical agents targeting the proteins involved in sensing and conducting DNA damage signals as well as other proteins in DNA repair pathways (Mateo et al., 2019; Cheng et al., 2022).

DNA damage sensor proteins are key functional proteins to initiate repair and can sense multiple DNA damage signals, in which poly (ADP-ribosyl) polymerase-1 (PARP-1) is widely recognized to be the primary responder to SSBs while it could also bind and signal DSBs (Li and Yu 2013; Ceccaldi et al., 2015; Mateos-Gomez et al., 2015; Pandey and Black, 2021). After rapidly detecting the DNA damage, PARP-1 synthesizes of poly (ADP-ribose) (PAR) chains on itself and many different proteins near the damage site initiating recruitment of DNA repair complexes. The formation of the PAR chain can promote the release of PARP-1 from the position where it binds to the damaged DNA so that the other repair proteins could contact with the damaged site. Inhibition of PARP will reduce the synthesis of PAR chains, making PARP unable to dissociate from damaged DNA, thus preventing the recruitment of other repair proteins (Zandarashvili et al., 2020). The failure of PAR chain formation and the release of PARP from damaged DNA will lead to the enrichment of SSBs, which can be transformed into single-sided DSBs during DNA replication (Bixel and Hays, 2015). However, in the cells with the absence of intact DSBs repair pathways, such as in BRCA1 and BRCA2 mutated cells, the persistent DSBs are toxic and even deadly. Many small molecule PARP inhibitors (PARPi) targeting the catalytic activity of PARP-1 are now approved and clinically used in patients with breast, ovarian, prostate, and pancreatic cancers deficient in other DDR components, and the expanded utilities of small molecule PARP inhibitors in other cancer types are under consideration (Ramakrishnan Geethakumari et al., 2017; Zimmer et al., 2018; Hammel et al., 2020; Xie et al., 2020).

For DSBs, lupus Ku autoantigen protein (Ku) and the Mre11-Rad50-Nbs1 complex (MRN) play important roles. Ku is a protein heterodimer composed of Ku70/Ku80, which takes part in the NHEJ pathway and binds to DNA DSBs (Chen et al., 2021). Upon recognition and binding of DSBs, Ku recruits the DNA-dependent protein kinase catalytic subunit (DNA-PKcs) that assists in classical NHEJ repair. A class of compounds has been developed that abrogates the Ku-DNA end binding activity, inhibits cellular NHEJ, and enhances the cellular activity of radiomimetic agents and IR (Gavande et al., 2020). The MRN complex has nuclease activity and can bind DNA, so it can participate in the initial detection and processing of DSB (Rupnik et al., 2008), which is dependent on the nuclease activity of Mre11, the central factor of the MRN complex with endonuclease activity and 3′–5′ exonuclease activity (Stracker and Petrini, 2011). Upon bound to the damaged position, MRN recruits the DNA-damage signaling kinase ataxia-telangiectasia mutated (ATM), activates it, and triggers a series of signaling events that drive HR repair (Uziel et al., 2003). A class of inhibitors has been developed to selectively block the nuclease activity of Mre11 and prevent DNA damage repair (Dupré et al., 2008).

DNA damage signaling proteins trigger multiple post-translational modifications and the assembly of protein complexes, which amplify and diversify the DNA damage signals. Initially, one of three phosphatidylinositol-3 kinase-related kinases (PIKKs): DNA-PKcs, ATM, or ATM- and Rad3-Related (ATR) is activated by phosphorylation in response to DNA damage (Woods and Turchi, 2013). DNA-PKcs, forming a heterotrimeric complex with Ku, are required for proper DSBs repair by NHEJ. Using its activated kinase activity after being bound to the DNA terminus, it will phosphorylate itself and other target proteins to coordinates the NHEJ pathway (Falck et al., 2005; Uematsu et al., 2007). Following the appearance of DSB, the MRN complex activates ATM, which then phosphorylates histone H2AX as the main kinase (Burma et al., 2001). ATM could also phosphorylate checkpoint kinase 2 (Chk2) and p53 to impact cell cycle regulation and cytotoxicity (Cheng and Chen, 2010; Smith et al., 2010). ATR participates in HR, NER, long-patch BER, postreplication repair, interstrand cross-link repair, and replication fork restart after its activation by replication protein A (RPA)-coated ssDNA (Cimprich and Cortez, 2008). Several small molecules targeting three PIKKs such as VX-984 and CC-115 for DNA-PKcs, AZD0156 for ATM, VX-970, and AZD6738 for ATR are currently in different stages of clinical trials (Munster et al., 2016; Foote et al., 2018; Pike et al., 2018; Timme et al., 2018; Gorecki et al., 2020). In addition, checkpoint kinase 1 (Chk1) and Chk2, protein kinases that lie downstream of ATR and ATM, have also been utilized as therapeutic targets for drug development (Jobson et al., 2009; King et al., 2014).

## 3 Nucleic Acid-Sensing Pathways Connect DNA Damage to Innate Immunity

**FIGURE 1:**
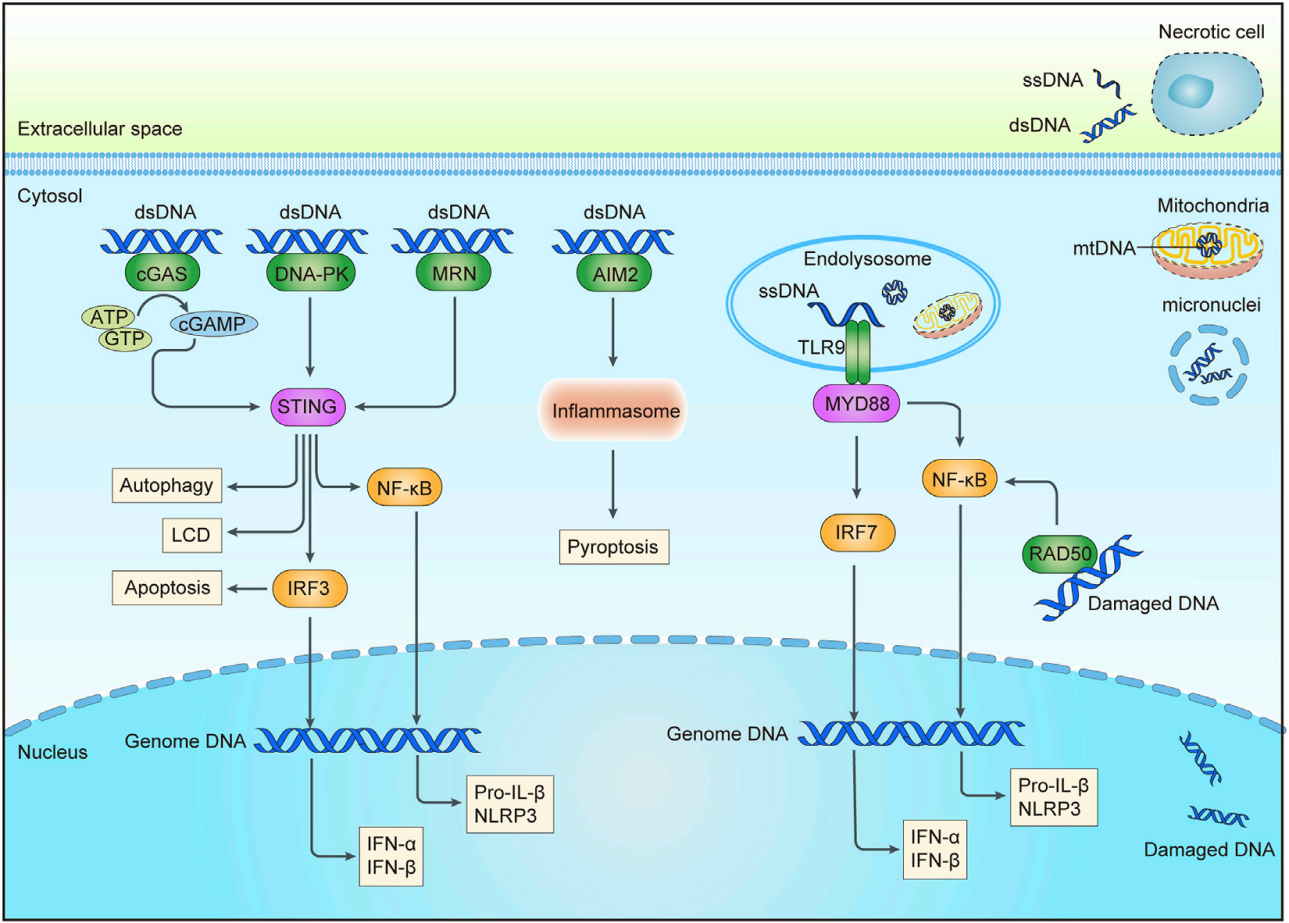
DNA sensing pathways triggered by DNA damage. An abnormal increase of intracellular DNA could come from the nucleus, micronuclei, or mitochondria after chemotherapy or radiation. DNA in endosomal may be from extracellular DNA of necrotic cells through endocytosis or cytoplasmic DNA through autophagy. Sensors for DNA are shown in green, including cGAS, DNA-PK, MRN, AIM2, and RAD50 in the cytoplasm, and TLR9 in the endolysosome. Adaptor molecules are shown in pink and downstream signaling molecules are shown in yellow. Activation of these pathways may result in the production of interferon (IFN) and other cytokines, apoptosis, pyroptosis, autophagy, etc., cGAS, cyclic GMP-AMP synthase; DNA-PK, DNA-dependent protein kinase complex; MRN, Mre11-Rad50-Nbs1 complex; AIM2, absent in melanoma 2; TLR9, Toll-like receptor 9; cGAMP, cyclic GMP-AMP; STING, stimulator of interferon genes; MYD88, myeloid differentiation primary response protein 88; IRF3/7, interferon regulatory factor 3/7; NF-κB, nuclear factor-κB; NLRP3, NOD-, LRR- and pyrin domain-containing 3; LCD, a lytic cell death program; dsDNA, double stranded DNA; ssDNA, single stranded DNA; mtDNA, mitochondrial DNA.

Cancer chemotherapy and radiotherapy aim to induce catastrophic DNA damage such as DSBs to cause cancer cell apoptosis, which can further aggravate the degree of DNA damage and promote the therapeutic effect when combined with DNA damage repair inhibitors. According to the severity of the DNA damage, some cancer cells directly initiate programmed cell death to clear the damaged genome beyond endurance. Besides, nucleic acid released from dying cells can activate the innate immune response of surrounding cells (Wang et al., 2021b). Even if DNA damage does not directly kill cells, increasing evidence indicates that the accumulation of nucleic acids in the cytoplasm caused by DNA damage can also trigger an inflammatory response within the cells (Nastasi et al., 2020). DNA damage-induced cytosolic nucleic acid shares common receptors (pattern recognition receptors, PRRs) and downstream effectors with those induced by viral or bacterial infections (Takeuchi and Akira, 2010; Taffoni et al., 2021), which are summarized below (Figure 1). And some agonists of these nucleic acid-sensing pathways are already in clinical trials (Table 1). Strikingly, recent research has indicated that the proteins that participated in DNA repair also play active roles in innate immune signaling.

**TABLE 1:** Summary of clinical trials of nucleic acid-sensing pathways-related agonists.

PRR	ClinicalTrials.gov Identifier	Agent(s)	Route of administration	Cancer type(s)	Clinical phase of development
STING	NCT04144140	E7766	Intratumoral	Lymphoma; advanced solid tumors	Phase 1/1b
STING	NCT04609579	SNX281 (or in combination with pembrolizumab)	Intravenous	Advanced solid tumor; advanced lymphoma	Phase 1
STING	NCT05070247	TAK-500 (or in combination with pembrolizumab)	Intravenous	Select locally advanced; metastatic solid tumors	Phase 1
TLR	NCT00960752	R848 gel (in combination with gp100 and MAGE-3 peptide vaccine)	Intradermally and subcutaneously	Melanoma	Phase 2
TLR	NCT02668770	MGN1703 (in combination with ipilimumab)	Subcutaneously and intratumoral injection	Advanced cancers; melanoma	Phase 1
TLR3	NCT03734692	Rintatolimod (in combination with cisplatin and pembrolizumab)	Intraperitoneal	Ovarian cancer recurrent	Phase 1; Phase 2
TLR7	NCT00899574	Imiquimod	Cream	Breast cancer; breast neoplasms	Phase 2
TLR7	NCT00941811	Imiquimod	Cream	HPV	Phase 2
TLR7	NCT01421017	Imiquimod (in combination with cyclophosphamide or radiotherapy)	Cream	Breast cancer; metastatic breast cancer; recurrent breast cancer	Phase 1; Phase 2
TLR7	NCT03416335	DSP-0509 (or in combination with pembrolizumab)	Intravenous	Neoplasms	Phase 1; Phase 2
TLR7	NCT04101357	BNT411 (or in combination with atezolizumab, carboplatin and etoposide)	Intravenous	Solid tumor; extensive-stage small cell lung cancer	Phase 1; Phase 2
TLR7	NCT04338685	RO7119929 (in combination with Tocilizumab)	Oral	Carcinoma; hepatocellular; biliary tract cancer; secondary liver cancer; liver metastases	Phase 1
TLR7	NCT04588324	SHR2150 (or in combination with chemotherapy plus PD-1 or CD47 antibody)	Oral	Solid tumor	Phase 1; Phase 2
TLR7/8	NCT00821652	Resiquimod (in combination with NY-ESO-1 protein vaccination)	Subcutaneously	Tumors	Phase 1
TLR7/8	NCT04278144	BDC-1001 (or in combination with nivolumabe)	Intravenous	HER2 positive solid tumors	Phase 1; Phase 2
TLR7/8	NCT04799054	TransCon (or in combination with pembrolizumab)	Intratumoral	Advanced solid tumor; locally advanced solid tumor; metastatic solid tumor	Phase 1; Phase 2
TLR7/8	NCT04840394	BDB018 (or in combination with pembrolizumab)	Intravenous	Advanced solid tumors	Phase 1
TLR8	NCT01294293	VTX-2337 (in combination with pegylated liposomal doxorubicin hydrochloride or paclitaxel)	Subcutaneously	Ovarian epithelial; fallopian tube; peritoneal cavity cancer	Phase 1
TLR8	NCT01334177	VTX-2337 (in combination with cetuximab)	Subcutaneously	Locally advanced; recurrent; metastatic squamous cell cancer of head and neck	Phase 1
TLR8	NCT01666444	VTX-2337 (in combination with pegylated liposomal doxorubicin)	Intravenous	Epithelial ovarian cancer; fallopian tube cancer; primary peritoneal cancer	Phase 2
TLR8	NCT01836029	VTX-2337 (in combination with chemotherapy and cetuximab)	Intravenous	Carcinoma; squamous cell of head and neck	Phase 2
TLR8	NCT03906526	VTX-2337 (or in combination with nivolumabe)	Subcutaneously or intratumoral injection	Carcinoma; squamous cell	Phase 1
TLR9	NCT00185965	CPG 7909 (in combination with radiation therapy)	Intratumoral	Recurrent low-grade lymphomas	Phase 1; Phase 2
TLR9	NCT02254772	SD-101 (in combination with ipilimumab and radiation therapy)	Intratumoral	Recurrent low-grade B-cell lymphoma	Phase 1; Phase 2
TLR9	NCT02927964	SD-101 (in combination with Ibrutinib and radiation therapy)	Intratumoral	Relapsed or refractory grade 1–3A follicular lymphoma	Phase 1; Phase 2
TLR9	NCT03410901	SD-101 (in combination with anti-OX40 antibody BMS 986178 and radiation therapy)	Intratumoral	Low-grade B-cell non-hodgkin lymphomas	Phase 1
TLR9	NCT03618641	CMP-001 (in combination with nivolumab)	Intravenous	Melanomal; lymph node cancer	Phase 2
TLR9	NCT03831295	SD-101 (in combination with anti-OX40 antibody BMS 986178)	Intratumoral	Advanced malignant solid neoplasm; extracranial solid neoplasm; metastatic malignant solid neoplasm	Phase 1
TLR9	NCT04050085	SD-101 (in combination with nivolumab and radiation therapy)	Intratumoral	Chemotherapy-refractory metastatic pancreatic cancer	Phase 1
TLR9	NCT04270864	Tilsotolimod (in combination with ipilimumab and nivolumab)	Intratumoral	Advanced cancer	Phase 1
TLR9	NCT04387071	CMP-001 (in combination with INCAGN01949)	Intratumoral	Stage IV pancreatic; other cancers except melanoma	Phase 1; Phase 2
TLR9	NCT04401995	Vidutolimod (in combination with nivolumab)	Subcutaneously and intratumoral injection	Melanoma	Phase 2
TLR9	NCT04708418	CMP-001 (in combination with pembrolizumab)	Subcutaneously and intratumoral injection	Operable melanoma	Phase 2
TLR9	NCT04935229	SD-101 (or in combination with nivolumab or ipilimumab)	Pressure-enabled hepatic artery infusion	Metastatic uveal melanoma in the liver	Phase 1
TLR9	NCT05220722	SD-101 (in combination with checkpoint blockade)	Pressure-enabled hepatic artery infusion	Hepatocellular carcinoma; intrahepatic cholangiocarcinoma	Phase 1; Phase 2

### 3.1 DNA Sensing Pathways

#### 3.1.1 Cyclic GMP-AMP Synthase

Normally, DNA is trapped in the nucleus and mitochondria and rapidly degraded by nucleases in the cytoplasm and endolysosomes. DNA-sensing receptors could detect increased amounts of intracellular DNA. Currently, cyclic GMP-AMP synthase (cGAS), a nucleotidyltransferase (NTase), is the most widely accepted dsDNA sensor that acts followed by stimulator of interferon genes (STING) performing multiple functions (Kuchta et al., 2009; Sun et al., 2013; Wu et al., 2013). cGAS normally resides to be inactive. Initial studies considered cGAS as a cytoplasmic protein, in which cGAS could not interact with nuclear or mitochondrial DNA, but some recent works indicate that cGAS also resides in the nucleus constitutively (Gentili et al., 2019; Jiang et al., 2019; Volkman et al., 2019). cGAS can be activated not only by viral or bacterial infection-related DNA entering the cytoplasm but also by endogenous self-DNA, including cytosolic DNA from nucleus and mitochondria, DNA in cytoplasmic micronucleus, and chromatin in the nucleus (Li X.-D. et al., 2013; West et al., 2015; Dou et al., 2017; Glück et al., 2017; Harding et al., 2017; Mackenzie et al., 2017; Gratia et al., 2019). Upon binding to DNA, cGAS assembles into a dimer at an active state and converts ATP and GTP into the second messenger cyclic GMP-AMP (cGAMP) (Ablasser et al., 2013; Diner et al., 2013; Gao et al., 2013; Zhang et al., 2013). The complete activation and stabilization of cGAS-DNA complexes require DNA lengths to exceed a certain threshold, allowing two or more cGAS molecules to bind to the same DNA to form oligomeric structures or condensates (Li X. et al., 2013; Zhang J.-Z. et al., 2014; Andreeva et al., 2017; Luecke et al., 2017; Du and Chen, 2018; Hooy and Sohn, 2018). The cyclic-dinucleotide sensor STING, an ∼40 kDa dimeric transmembrane protein located in the endoplasmic reticulum (ER), could detect and bind to cGAMP to make a conformational change, which results in its translocation from the ER to the Golgi apparatus and the activation of TANK-binding kinase 1 (TBK1) (Ishikawa and Barber, 2008; Burdette et al., 2011; Liu et al., 2015; Ergun et al., 2019; Shang et al., 2019; Zhang et al., 2019; Zhao et al., 2019). The activated TBK1 phosphorylates itself, STING, and the interferon regulatory factor 3 (IRF3), after which the active IRF3 dimer is transported to the nucleus to activate the expression of type I interferon genes. The cGAS-STING signaling can also lead to the transcription of pro-inflammatory cytokines-related genes *via* nuclear factor-κB (NF-κB) (de Oliveira Mann et al., 2019). In addition, STING is related to autophagy induction, however, its functional mechanism still remains to be elucidated (Watson et al., 2012). Growing studies suggest that the activation of STING can trigger cell death by various means. For example, STING could facilitate respective programmed cell death by inducing the production of many pro-apoptotic and pro-necroptotic molecules (Paludan et al., 2019). Furthermore, the accumulation of lysosomal STING could trigger lysosome membrane permeabilization, leading to the release of lysosomal hydrolases with cell death as a result (Gaidt et al., 2017). Besides, phosphorylated IRF3 downstream of STING can stimulate apoptosis by reducing the Bcl-xL-dependent suppression of the permeability of mitochondrial outer membrane in mitotic cells (Zierhut et al., 2019; Xie et al., 2021).

#### 3.1.2 Toll-Like Receptors 9

Toll-like receptors (TLRs) are important components of innate immune responses induced by pathogenic microorganisms or tissue injury (Moresco et al., 2011). TLR, a highly conserved intracellular transmembrane protein, has emerged as a key PRR in the past 20 years. It exists in multiple types of cells, such as T cells, B cells, APC, epithelial cells, and endothelial cells (Chang 2010). All TLRs possess a leucine-rich-repeat (LRR) domain binding ligand extracellularly, a transmembrane domain, and a cytosolic Toll/IL-1 receptor (TIR) homology domain (Delneste et al., 2007). In all TLRs, TLR9 is found specifically in the endosomes and can be activated by single-stranded DNA containing unmethylated cytidine-phosphate-guanosine (CpG) dinucleotides escaping from the digestion of nucleases such as DNase II (Kumagai et al., 2008). TLR9 must undergo proteolytic processing in the endosomal compartments to complete ligand-mediated dimerization and activation (Majer et al., 2017). TLR9 not only triggers plasmacytoid dendritic cells to produce type I IFN and activate the polyclonal B cells through the myeloid differentiation primary response protein 88 (MYD88) and interferon-regulatory factor 7 (IRF7) signaling pathway, but also induces the production of inflammasome-related factors pro-interleukin-1β (pro-IL-1β) and NOD-, LRR- and pyrin domain-containing 3 (NLRP3) *via* NF-κB (Honda et al., 2005; Zhang X. et al., 2014). According to a recent study, TLR9 can sense mitochondrial DNA during mitophagy, and induce C-X-C motif chemokine ligand 10 (CXCL10) expression and CD8^+^ T cell recruitment, which reveals a novel role of chemotherapy in the innate immune response (Limagne et al., 2022).

#### 3.1.3 Absent in Melanoma 2

The absent in melanoma 2 (AIM2), containing pyrin and HIN domains, takes an important part in inflammasome activation as the dsDNA-sensing receptor in the cytoplasm of cells (Fernandes-Alnemri et al., 2009; Hornung et al., 2009). The N-terminal pyrin domain (PYD) of AIM2 could interact with apoptosis-associated speck-like protein (ASC), which possesses a caspase recruitment domain (CARD) and a C-terminal HIN domain sensing cytoplasmic DNA (Hornung et al., 2009; Wang and Yin, 2017). The recruitment of ASC leads to the generation of fibrilar super-structures, to which Caspase-1 is bound by CARD-CARD interactions (Miao et al., 2011; Lugrin and Martinon, 2018). The oligomerization of ASC leads to the activation of some proteins such as the conversion of pro-IL-1β into the biologically active IL-1β. The inflammasome can also trigger pro-inflammatory pyroptosis (Liang et al., 2020). Gasdermins family proteins are the important factors mediating inflammatory cell death (Kovacs and Miao, 2017), in which Gasdermin D (GSDMD) can be cleaved by Caspase-1 to release the N-terminal of Gasdermin so that it can polymerize and cause perforation of the plasma membrane, allowing the intracellular substance to leak out, causing cell death (Lammert et al., 2020). It is well known that AIM2 can detect dsDNA in the cytosol, but recent studies have found that AIM2 can also sense DSBs directly within the nucleus to induce intestinal epithelial cells and bone marrow cells to initiate the caspase-1-dependent death (Hu et al., 2016).

#### 3.1.4 DNA-PK Complex

Recent findings reveal that some proteins in DNA repair also take part in the sense of foreign DNA in the cytosol. DNA-dependent protein kinase (DNA-PK) complex assembled from Ku70/Ku80 heterodimer and the kinase subunit (DNA-PKcs) can sense DNA DSBs in response to repair the DNA damage in the NHEJ pathway, and Ku has also been reported to detect viral DNA in human cells as a PRR to induce type I and type III interferons or pro-inflammatory cytokines (Abe et al., 2019). DNA stimulates the Ku complex to translocate into the cytoplasm and bind to the dsDNA terminals using its middle domain independent of DNA sequence (Sui et al., 2021a; Sui et al., 2021b). Numerous studies have reported that STING is the downstream adaptor of Ku to induce type I/III interferons and inflammatory cytokines *via* phosphorylation of IRF3 (Sui et al., 2017). Ku complex is abundantly expressed in aged human and mouse CD4^+^ T cells, and it recognizes accumulating cytoplasmic DNA in the cytoplasm, which facilitates the recruitment of DNA-PKcs and phosphorylation of the kinase ZAK, ultimately promoting the proliferation and activation of CD4^+^ T cells (Wang et al., 2021a).

#### 3.1.5 Mre11-Rad50-Nbs1 Complex

MRN complex consisting of two MRE11 subunits, two RAD50 units, and two NBS1 subunits, can sense and respond to DNA damage firstly in DDR to orchestrate DDR response in DSBs and replication fork collapse (Zhu et al., 2018; Tisi et al., 2020). The hetero-hexamers MRN complex could conduct ATP hydrolysis of RAD50, bind multiple DNA molecules, and link DNA molecules with exonuclease and endonuclease (Kondo et al., 2013). RAD50 could recognize cytosolic dsDNA and bind caspase-recruitment domain (CARD9), a pro-inflammatory signaling adaptor, to its zinc-hook region, which tends to recruit Bcl-10, leading to the activation of NF-κB and the generation of pro-inflammatory cytokine IL-1β (Roth et al., 2014).

### 3.2 RNA Sensing Pathways

#### 3.2.1 Toll-Like Receptor 3/7/8

**FIGURE 2:**
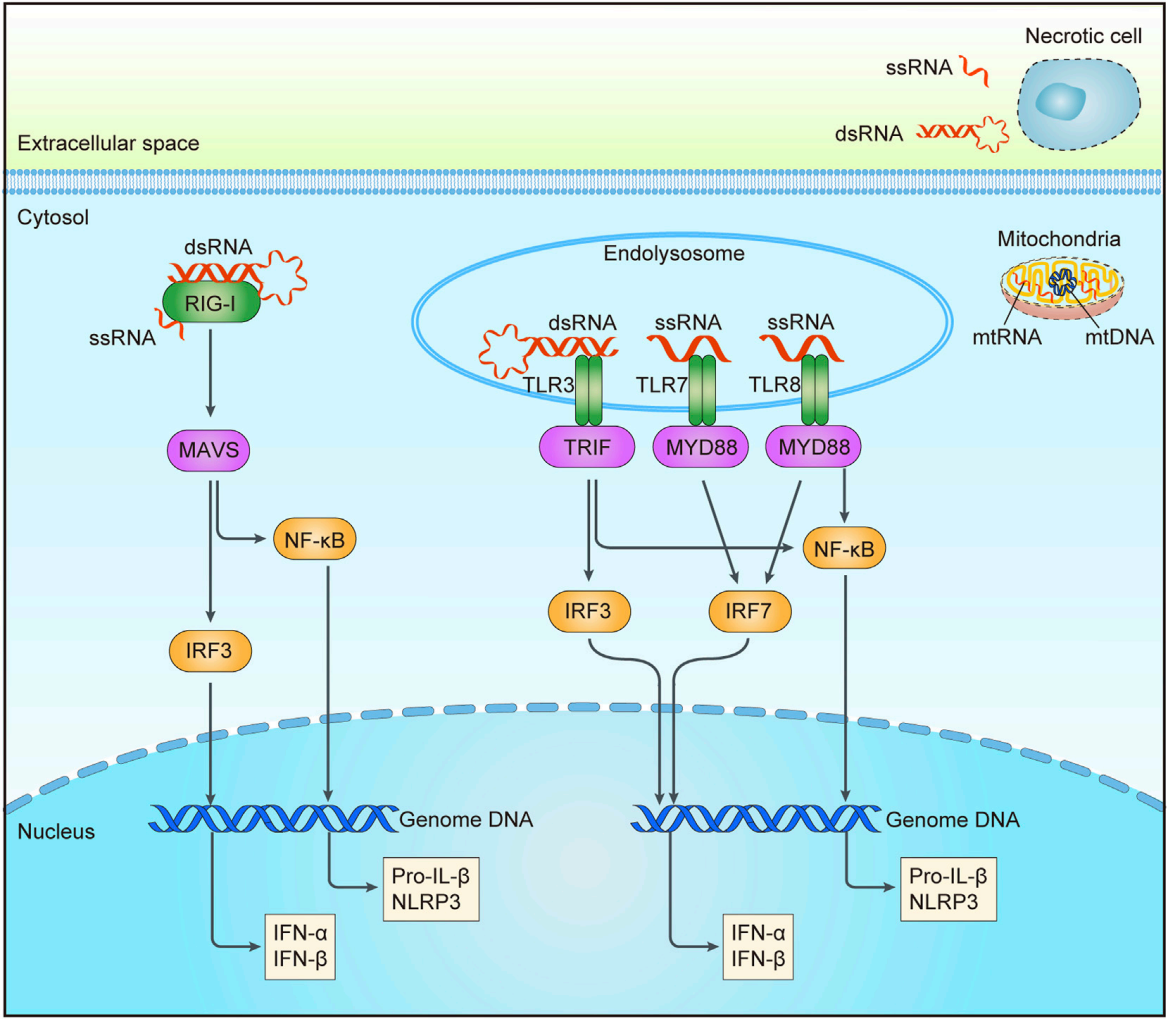
RNA sensing pathways triggered by DNA damage. Aberrant increase of intracellular RNA could be from mitochondria after chemotherapy or radiation. RNA in endosomal may be from extracellular RNA of necrotic cells through endocytosis or cytoplasmic RNA through autophagy. Sensors for RNA are shown in green, including RIG-1 in the cytoplasm, and TLR3/7/8 in the endolysosome. Adaptor molecules are shown in pink and downstream signaling molecules are shown in yellow. Activation of these pathways may result in the production of interferon (IFN) and other cytokines, etc. RIG-I, a retinoic acid-inducible gene I; TLR3/7/8, Toll-like receptor 3/7/8; MAVS, mitochondrial antiviral signaling protein; TRIF, Toll-like receptor adaptor molecule 1; MYD88, myeloid differentiation primary response protein 88; IRF3/7, interferon regulatory factor 3/7; NF-κB, nuclear factor-κB; dsRNA, double stranded RNA; ssRNA, single stranded RNA; mtRNA, mitochondrial RNA; mtDNA, mitochondrial DNA.

In mammalian cells, both single-stranded and double-stranded RNA can be recognized by PRR as pathogen-associated molecular patterns (PAMPs) or damage-associated molecular patterns (DAMPs) (Figure 2). RNA from necrotic cells may be internalized by cells *via* clathrin-dependent endocytosis, or it can enter cells after complexation with peptides or within immune complexes (Lövgren et al., 2004; Barrat et al., 2005; Itoh et al., 2008; Ganguly et al., 2009). The activation of TLR, a type I transmembrane protein, is required for inducing innate and adaptive immune responses, among which TLR3 can recognize double-stranded RNA and structured RNA containing a partial stem in secondary structures of single-stranded RNA, and TLR7/8 can recognize the fragments of single-stranded RNA (Alexopoulou et al., 2001; Karikó et al., 2004; Cavassani et al., 2008; Tatematsu et al., 2013). Depending on the intracellular compartments to recognize ligands, discriminate self-and non-self-derived nucleic acids, TLRs activate the downstream signaling pathways (Gay et al., 2014). Upon ligand binding, the dimers of TLRs will emerge after binding ligands to recruit the cytosolic adaptor proteins with TIR domain, such as MyD88 and Toll-like receptor adaptor molecule 1 (TRIF) (Yamamoto et al., 2003; Vyncke et al., 2016). The recruitment of MyD88 could activate NF-κB through type I interferon induction by IRF7 and tumor necrosis factor receptor-associated factor 6 (TRAF6). Intriguingly, among the TLRs, only TLR3 could signal to induce the generation of type I interferon and proinflammatory cytokines by the recruitment of TRIF rather than depending on MyD88 (Oshiumi et al., 2003).

#### 3.2.2 (RIG-I)-Like Receptors

RLRs are composed of retinoic acid-inducible gene I (RIG-I), melanoma differentiation-associated protein 5 (MDA5), and laboratory of genetics and physiology2 (LGP2), which can help the innate immune system sense cytosolic RNA (Kang et al., 2004; Yoneyama et al., 2004; Yoneyama et al., 2005; Ori et al., 2017). RIG-I and MDA5 contain two CARDs at the N-terminus, a DExD/H box RNA helicase domain in the central and a C-terminal domain (CTD), which mediate downstream signaling by sensing RNA. MDA5 preferentially recognizes dsRNA longer than 1 kb unlike RIG-I, which could bind relatively short dsRNA (Kato et al., 2008; Goubau et al., 2014). The CARD domains of RIG-I and MDA5 oligomerize after binding to dsRNA and tend to interact with the CARD of mitochondrial antiviral signaling protein (MAVS) (Kawai et al., 2005; Seth et al., 2005). Then the oligomerization of MAVS occurs after binding to RIG-I or MDA5 to form prion-like aggregates, upon which the downstream signaling pathways can be activated (Hou et al., 2011). Besides, MAVS could trigger the transcription of type I IFNs *via* IRF3 or IRF7 by activating TBK1, and the transcription of inflammatory cytokines *via* NF-κB by activating the IKK complex (IKKα, IKKβ, NEMO) (Goubau et al., 2013; Ori et al., 2017).

Recent research has found that the DNA fragments released from ionizing radiation-induced double-strand DNA breaks could activate both the cGAS/STING-dependent DNA-sensing pathway and the MAVS-dependent RNA sensing pathway (Feng et al., 2020). Interestingly, chemotherapeutic agents and ionizing radiation can also lead to mitochondrial DNA double-strand breaks (mtDSBs). After mtDNA breaks, BAX and BAK mediate herniation, then the mitochondrial RNA will be released into the cytoplasm, triggering the RIG-I-MAVS-dependent immune response (Tigano et al., 2021). These studies suggest that DNA damage can also activate innate immune responses through RNA sensing pathways.

## 4 The Impacts of Nucleic Acid-Sensing Pathways on DNA Repair

### 4.1 Cyclic GMP-AMP Synthase-Stimulator of Interferon Genes

As mentioned above, several DDR proteins, such as DNA-PK and MRN complex, can not only can participate in the DNA repair, but also take part in the onset of inflammatory responses. Conversely, PRRs, first discovered to sense immune-stimulatory nucleic acids, have also been found to take part in the regulation of DDR. Previously, cGAS-STING signaling was simply identified as a response pathway to cytosol dsDNA, however, they have recently been found to function in DNA repair, independent of interferon response. cGAS could inhibit the repair of DNA DSBs by HR without relying on STING and the catalytic activity of cGAS (Hopfner and Hornung, 2020). There are two mechanisms for cGAS-dependent HR inhibition. In one of them, because it has been found at the sites of chromosomal damage marked by PARP1 and γ-H2AX, cGAS could prevent the recruitment of proteins necessary for the HR process by interacting with γ-H2AX and PAR (Liu et al., 2018). On the other hand, subsequent work suggested that cGAS could prevent RAD51-DNA filaments to pair and the broken DNA strand from invading into the homologous strand by binding the homologous dsDNA template to form oligomeric clusters (Jiang et al., 2019). However, the detailed mechanism remains to be investigated further.

STING has been confirmed to promote DDR and enable cell survival without cGAS, though they are partners (Cheradame et al., 2021). Downregulation of STING increases cell death and makes breast cancer cells more sensitive to genotoxic treatment. Following chemotherapy regimens, some STINGs are found to be located at the inner nuclear membrane and bind the NHEJ proteins DNA-PKcs, Ku70, and Ku80, suggesting that STING may control NHEJ-mediated DNA repair by cooperating with DNA-PK (Ferguson et al., 2012; Morchikh et al., 2017; Sui et al., 2017; Cheradame et al., 2021). Regrettably, its specific mechanism remains unclear.

Recent research has proposed a novel mechanism that IR-induced DNA damages would trigger the phosphorylation and activation of phosphoribosyl pyrophosphate synthetases PRPS1/2 *via* ATM and cGAS/STING/TBK1, which could promote the synthesis of deoxyribonucleotide given that the PRPSs are the rate-limiting enzymes. Then the increased deoxyribonucleotide will help for DNA repair. Nevertheless, it remains to be clarified how the cells respond to DNA damage *via* the cGAS-STING pathway under different contexts (Kornberg et al., 1955; Hove-Jensen, 1988; Liu et al., 2021).

### 4.2 Toll-Like Receptors

TLRs are the key members of the innate immune system, functioning as the first line of defense against multiple injurious substances (Trinchieri and Sher, 2007; Barton and Kagan, 2009; Kawai and Akira, 2011). Recent literature has shown that activation of TLRs could also promote DNA repair by upregulating the expression of DNA repair genes, apart from upregulating cellular defense systems. Upon TLR9 stimulation, there is a significant increase of mRNAs to participate in adjusting cell cycles and DNA repair after CpG DNA is injected into the abdominal cavity (Zheng et al., 2008; Klaschik et al., 2010; Sommariva et al., 2011). And NER gene expression is increased by treating bone marrow-derived cell lines with the TLR7/8 agonist (Imiquimod) *in vitro* (Fishelevich et al., 2011). It has been deduced that TLR signaling pathways may result in transcriptional activation of DNA repair machinery through direct and indirect mechanisms. The promoter regions of many genes involved in DNA repair contain the binding sites of activator protein-1 (AP-1) (Xiao et al., 1993; Zhong et al., 2000). The transcriptional control of DNA repair might be linked to TLR agonist treatment through the transcriptional activation function of the AP-1. On the other hand, DNA repair can be promoted because the activated TLR could induce the generation of cytokines. The cytokines such as IL-12 may be sensed by their appropriate cytokine receptor, leading to the increased transcription levels of DNA repair genes (Majewski et al., 2010). However, increased DNA repair is detrimental for cancer treatment. The previous study has found that TLR9 agonist treatment upregulates the genes associated with DNA repair in immune cells but downregulates them in tumor cells, which is contributed to the death of cancer cells (Sommariva et al., 2011). Those conflicting data highlight the need for further research into how immune versus stromal cells, and normal versus cancerous cells respond to TLR agonists, as well as related DNA repair and cell survival.

### 4.3 AIM2-Like Receptors

AIM2-like receptors (ALRs) are a large family of structurally related proteins that are generally considered to act as intracellular DNA sensors alerting the innate immune system. Recent studies reveal that ALRs perform different functions outside the immune system. It has been demonstrated that DNA breaks are repaired more efficiently in mice and cells lacking ALRs (ALR^−/−^ mice lack the entire ALR locus containing all 13 ALR genes on Chromosome 13), resulting in the stronger resistance to the genotoxic effects of irradiation and chemotherapy (Brunette et al., 2012; Gray et al., 2016; Jiang et al., 2021). Mechanistically, nuclear ALRs limit DNA repair machinery access to damaged sites by binding the chromatin, and self-oligomerization promoted chromatin compaction. These findings reveal that ALRs could be the possible target for new interventions against genotoxic tissue injury, but more research into the different members of the ALR family is needed.

## 5 Conclusion and Future Perspective

Targeting DDR factors in tumors has achieved outstanding success over the last decade, especially PARP inhibitors for cancer therapy. Recently, we have gained an improved understanding of the molecular mechanism of how DNA damage is interconnected to cellular innate immunity, which plays essential roles in the therapeutic efficacy of DNA repair targeted treatments (Pantelidou et al., 2019). Damaged nucleic acids also shape adaptive immune responses by activating innate immune cells. Accumulation of nucleic acids from necrotic cells can induce type I IFNs and other immune-regulatory cytokines production from bystander cells [e.g., dendritic cells (DCs)] to promote antitumor immunity through nucleic acid-sensing pathways. The DCs activated by the type I IFNs secretion will be transported to tumor-draining lymph nodes and cross-prime naïve CD8^+^ T lymphocytes (Ellermeier et al., 2013; Duewell et al., 2014; Klarquist et al., 2014; Woo et al., 2014). The cancer-induced host response and tumor rejection relies heavily on the immunological responses to danger DAMPs signals.

Immune checkpoint blockade (ICB), as a promising therapeutic strategy, has made tremendous strides in recent years providing an alternative to irradiation therapy or traditional chemotherapies. Unfortunately, the efficacy of ICB is limited to only a subgroup of cancer patients (depending on the type of the tumor), with an overall response rate of about 20% for all malignancies to date (Hargadon et al., 2018; Chowell et al., 2021). ICB is effective in “hot” tumors with T cell infiltration rather than in “cold” tumors lacking T cell infiltration (Petitprez et al., 2020). Novel strategies for activating innate immunity within the TME to promote the antitumor immune responses have emerged in recent years, with the goal of eradicating the disease in “cold” tumors (Iurescia et al., 2018). Agonists of the nucleic acid-sensing pathways (such as cGAMP, agonists of STING) have been applied to eradicate tumor mass and induce a durable anti-tumor immune response (Chin et al., 2020; Pan et al., 2020). However, since some PRRs are involved in DDR and tumorigenesis, the impact of nucleic acid-sensing pathways on DNA repair cannot be ignored in therapeutic strategies aiming at promoting the activation of nucleic acid-sensing pathways.

As DDR inhibitors can trigger innate immune responses, DDR inhibition could be effective in combination with ICBs. The DDR inhibitors that target PARP have been investigated the most in anticancer immunotherapies. PARP inhibition could increase CD8^+^ T-cell infiltration and IFN-γ generation in tumors, and promote the tumor regression when used accompanied with anti-PD-1 antibody (Pantelidou et al., 2019; Shen et al., 2019). Recently, many other regents inhibiting DDR components have been developed and used preclinically (Cleary et al., 2020). DDR inhibition strategies combined with other therapeutic strategies possess tremendous potential to improve the effectiveness of cancer treatment because of their immunomodulatory effect on radiation and chemotherapies and immune checkpoint blocking. Furthermore, in order to kill tumor cells more precisely, innovative approaches (such as nanoparticles, viral particles, and targeted deliveries) beneficial to the precise delivery of a chemotherapeutic drug, DDR inhibitors, or nucleic acid-sensing pathways agonists to tumors can be used to induce local specific antitumor immune responses, which could significantly expand the therapeutic window for their use in cancer immunotherapies (Gentili et al., 2015; Wilson et al., 2018; Wang et al., 2020; Xu et al., 2021).
